# Evaluation of women’s aging influence on sperm passage inside the fallopian tube using 3D dynamic mechanical modeling

**DOI:** 10.3389/fbioe.2024.1324802

**Published:** 2024-04-12

**Authors:** Mayssam Nassir, Mattan Levi, Amir Wiser, Natan T. Shaked

**Affiliations:** ^1^ Department of Biomedical Engineering, Faculty of Engineering, Tel Aviv University, Tel Aviv, Israel; ^2^ Faculty of Medicine, Tel Aviv University, Tel Aviv, Israel

**Keywords:** fallopian tube, sperm transportation, 3D reconstruction, women’s aging, infertility, finite element modelling, biomechanical simulation

## Abstract

The fallopian tubes play an important role in human fertility by facilitating the spermatozoa passage to the oocyte as well as later actively facilitating the fertilized oocyte transportation to the uterus cavity. The fallopian tubes undergo changes involving biological, physical, and morphological processes due to women aging, which may impair fertility. Here, we have modelled fallopian tubes of women at different ages and evaluated the chances of normal and pathological sperm cells reaching the fertilization site, the ampulla. By utilizing a unique combination of simulative tools, we implemented dynamic three-dimensional (3D) detailed geometrical models of many normal and pathological sperm cells swimming together in 3D geometrical models of three fallopian tubes associated with different women’s age groups. By tracking the sperm cell swim, we found that for all age groups, the number of normal sperm cells in the ampulla is the largest, compared with the pathological sperm cells. On the other hand, the number of normal sperm cells in the fertilization site decreases due to the morphological and mechanical changes that occur in the fallopian tube with age. Moreover, in older ages, the normal sperm cells swim with lower velocities and for shorter distances inside the ampulla toward the ovary. Thus, the changes that the human fallopian tube undergoes due to women’s aging have a significant influence on the human sperm cell motility. Our model of sperm cell motility through the fallopian tube in relation to the woman’s age morphological changes provides a new scope for the investigation and treatment of diseases and infertility cases associated with aging, as well as a potential personalized medicine tool for evaluating the chances of a natural fertilization per specific features of a man’s sperm and a woman’s reproductive system.

## 1 Introduction

The fallopian tubes, also known as uterine tubes, are important parts of the female reproductive system and have a major effect on human fertility. The fallopian tubes are a pair of muscular tubes leading from the ovaries of the human reproductive system into the uterus. The function of the fallopian tubes is to facilitate the passage of the sperm cells to the oocyte and afterward to actively facilitate the fertilized oocyte transportation to the uterus cavity (Eddy and Pauerstein, 1980). The fallopian tubes provide a proper environment for spermatozoa and oocyte transportation, and they can be affected by a wide range of factors and conditions that may impair fertility (Talukdar and Sahu, 2016). Due to their significant influence on fertility, fallopian tubes are gaining importance in clinical studies and have become an interesting subject of research in the last decade (Hunter, 1998; Briceag et al., 2015; Dulohery et al., 2020; Vitale et al., 2022). The fallopian tubes undergo changes involving biological, physical, and morphological processes due to women’s aging. With age, the cellular senescence of fallopian tubes is affected and may contribute to a progressive decline in reproductive capacity (Shirasuna and Iwata, 2017). Therefore, several studies described the changes in morphology, cilia activity, and main functions of the fallopian tube in different women age groups, aiming to be utilized for the investigation and treatment of diseases and infertility associated with aging (Barros et al., 2002; Talukdar and Sahu, 2016; Devi et al., 2017; Veerraju and Sesi, 2019).

The fallopian tube is a hollow seromuscular organ attached distally to the ovary and proximally to the lateral aspect of the uterine fundus (Eddy and Pauerstein, 1980). The fallopian tube is composed of four regions: intramural, isthmus, ampulla, and infundibulum. The intramural region connects the fundus of the uterus with the isthmus. The isthmus is a short and less densely ciliated tube with fewer mucosal folds. The ampulla is the longer portion of the fallopian tube and has highly ciliated longitudinal mucosal folds. In the ampulla region, the fertilization of the ovum by a sperm cell and the initial embryo development occur. The infundibulum is the proximal part of the fallopian tube near the ovaries. This region includes finger-like fimbria, which catch the released oocyte from the ovary during each menstrual cycle (Vue et al., 2018; Winuthayanon and Li, 2018; Narang et al., 2019). The fallopian tube length averages 10–12 cm and has a lumen diameter of less than 1 mm (Han et al., 2013; Szmelskyj, 2015; Narang et al., 2019). The wall of the fallopian tube is particularly composed of a mucosal layer. The mucosal layer forms multiple and branched longitudinal mucosal folds and is lined with a simple columnar epithelium consisting of morphologically and functionally different epithelial cells (Allen, 2009; Varga et al., 2018). Epithelium morphology enhances hydrodynamic interactions, impacting sperm function (Eisenbach and Giojalas, 2006; Suarez and Pacey, 2006). The interaction of sperm with the surface potentially guides the sperm cells geometrically, improving their chances of survival, promoting capacitation, and affecting fertilization rates (Smith and Yanagimachi, 1990; Timothy Smith and Nothnick, 1997; Ertan Kervancioglu et al., 2000). However, these studies used flat solid surfaces that do not accurately represent the real morphology and mechanical properties of the lumen of the fallopian tube. Several studies have utilized droplet microfluidics to produce soft curved interfaces with controlled shapes and sizes to examine sperm behavior in real environment parameters. In a previous study, droplet microfluidics was utilized to investigate sperm behavior at soft curved interfaces, with individual sperm enclosed in droplets (Raveshi et al., 2021). The study found that different surface curvature geometry induced different sperm motility modes. These results emphasize the importance of changes in mammalian fallopian tube geometry in guiding sperm towards the site of fertilization or affecting the fertilization success rate (Raveshi et al., 2021). The fallopian tube is crowded with secretory and ciliated cells with hair-like structures, the cilia, where each cilium is about 10 µm long and 0.25 µm in diameter (Satir, 1992; Lyons et al., 2006). The fallopian tube surface has cyclic peristaltic contractions, which generate a sinusoidal wave and the swaying motions of the cilia tips (Croxatto, 2002; Lyons et al., 2006). The cilia of the fallopian tube assist self-propulsion of sperm cells toward the ampulla, fertilization site, in the ovulation period and transport the developing embryo to the uterine body if fertilization occurs (Blake, 1982; Coy et al., 2012; Yuan et al., 2021). Theoretical models of the fallopian tube fluid dynamics in a narrow tube have been developed (Ashraf et al., 2016; Akbar et al., 2017; Hayat et al., 2017; Zeeshan et al., 2017; Ahmadmawlood and Malathsagbanabied, 2019). A previous study presented a mathematical model analyzing the peristaltic-ciliary flow of third-grade fluid within the fallopian tubal (Ahmadmawlood and Malathsagbanabied, 2019). Observations conducted in the natural environment of the fallopian tube reveal that the movement of sperm plays a crucial role in transporting them to the central region of the fallopian tube lumen. While sperm motility is vital for this initial transport, it is not the primary factor influencing the overall progress of sperm within the female genital tract (Kölle et al., 2009, 2010; Kölle, 2012). Previous research has demonstrated that sperm cells move forward due to the strong current produced by ciliary beating and smooth muscle contraction, particularly intensified during ovulation (Kodithuwakku et al., 2007; Kölle et al., 2009; Ikawa et al., 2010; Okabe, 2015). Consequently, the coordinated action of the fallopian tube’s ciliary-peristaltic movements and the dynamic flow of tubal fluid, along with sperm motility, facilitates the upward movement of sperm. This intricate interplay ultimately leads to the complete acquisition of sperm fertilizing capacity and sets the stage for successful fertilization within the fallopian tube. An additional factor affecting sperm motility is the ion signaling that is crucial for sperm adaption. Mammalian sperm relies on interpreting signals from the female reproductive tract and egg layers for successful fertilization. Recent studies used electrophysiological recordings and genetic models to confirm the importance of ion channels like CatSper in sperm function, offering potential targets for contraceptives and infertility treatments (Wang et al., 2021). Understanding defective sperm function, especially genetic causes, is crucial for infertility treatment and assessing patient and offspring health (Wang et al., 2021). Several studies described two-dimensional models of peristaltic flow, ciliary flow, flow of viscoelastic and non-Newtonian fluid to demonstrate the transport pathway of the spermatozoa and the fertilized oocyte within the fallopian tube fluid (Ashraf et al., 2016; Akbar et al., 2017; Hayat et al., 2017; Zeeshan et al., 2017). However, the transportation mechanism of sperm cells within peristaltic-ciliary flow through the fallopian tube is not completely understood and still unclear. Fallopian tubes change their morphology with aging, and exhibit the greatest changes between the reproductive and the postmenopausal age groups (Hwang and Song, 2004). Various studies observed important age-related variations of the fallopian tube while the length, lumen area, mucosal layer area, and outer diameter of the fallopian tube change between pre-reproductive- (0–14 years), reproductive- (15–44 years) and postmenopausal- (+45 years) age groups (Kim-Bjorklund et al., 1991; Hwang and Song, 2004; Talukdar and Sahu, 2016; Veerraju and Sesi, 2019). The length, the number, and the degree of convolution of the fallopian tube decrease with aging (Hwang and Song, 2004). Moreover, the total areas of the tube, mucosal layer, and lumen in the ampulla decrease in cross-section due to women aging (Talukdar and Sahu, 2016; Devi et al., 2017; Veerraju and Sesi, 2019). The mucosal layer of the fallopian tube is columnar at the reproductive stage due to strong muscular activity, but it changes into cuboidal or squamous with aging (Lisa et al., 1954; Wolman, 2014; Devi et al., 2017). Previous studies show a decreased number of tubal ciliated cells in the fallopian tube associated with age and strikingly appeared in the ampulla region (Li et al., 2013; Tao et al., 2020). The reduction of tubal ciliated cells may be due to reduced capacity for removing follicular fluid-induced genotoxicity within the fallopian tube (Coan et al., 2018). Additional studies indicate that the mechanical features of the fallopian tube tissue, as well as other biological tissues, are age dependent. The elasticity of the fallopian tube tissue decreases with aging, as well as the stiffness (Barros et al., 2002; Shirasuna and Iwata, 2017; Şendur et al., 2020; Jafarbeglou et al., 2021). Thus, the morphology and mechanical properties of the human fallopian tubes change in the different age groups.

In the current study, using a unique combination of simulative tools, we developed a mechanical model of sperm-cell swimming behavior within the human fallopian tube in different age groups and analyzed the women’s aging effects on the chances of normal and pathological sperm cells reaching the fertilization site. We used the 3D detailed geometrical models of the normal and pathological sperm cells developed in our previous studies (Nassir et al., 2022; 2023), but simplified the 3D geometry of the sperm models, including the external sperm cell real organs (head, flagellum, and midpiece) to allow dynamic 3D simulation of many sperm cells swimming together in the woman’s body. The pathological sperm models include frequent defects in the sperm head and flagellum morphologies appearing in WHO-2021 guidelines (World Health Organization, 2021). We then developed three 3D models of the human fallopian tubes, based on human uterus ultrasound and MRI results (Rimbach et al., 1998; De Geyter et al., 2007; La Parra Casado et al., 2013; Revzin et al., 2020; Luo et al., 2023). Each 3D geometrical model presented the human fallopian tube of a different women’s age group, and was built based on the morphological changes of the fallopian tube due to women’s aging, as reported in previous studies (Kim-Bjorklund et al., 1991; Hwang and Song, 2004; Talukdar and Sahu, 2016; Devi et al., 2017; Veerraju and Sesi, 2019). We applied peristalsis-ciliary activity along the inner walls of the different fallopian tube models. Dynamic simulations were performed for the normal and pathological sperm cells swimming inside the different fallopian tubes, evaluating the effects of the women aging on the chance for success of the sperm cells in reaching the fertilization site. We calculated the numbers of the sperm cells that succeeded in reaching the fertilization site inside the fallopian tube as a function of displacement and time. We also compared the simulation results of swimming behaviors inside the different fallopian tubes and estimated the women’s aging influence on the chances of the sperm cells chances of reaching the egg. Our advanced model shows an important age relation between the fallopian tube and the potential fertility of different age groups and may help in various disease conditions of the fallopian tube.

## 2 Methods and materials

### 2.1 3D modelling of the human fallopian tubes

**FIGURE 1:**
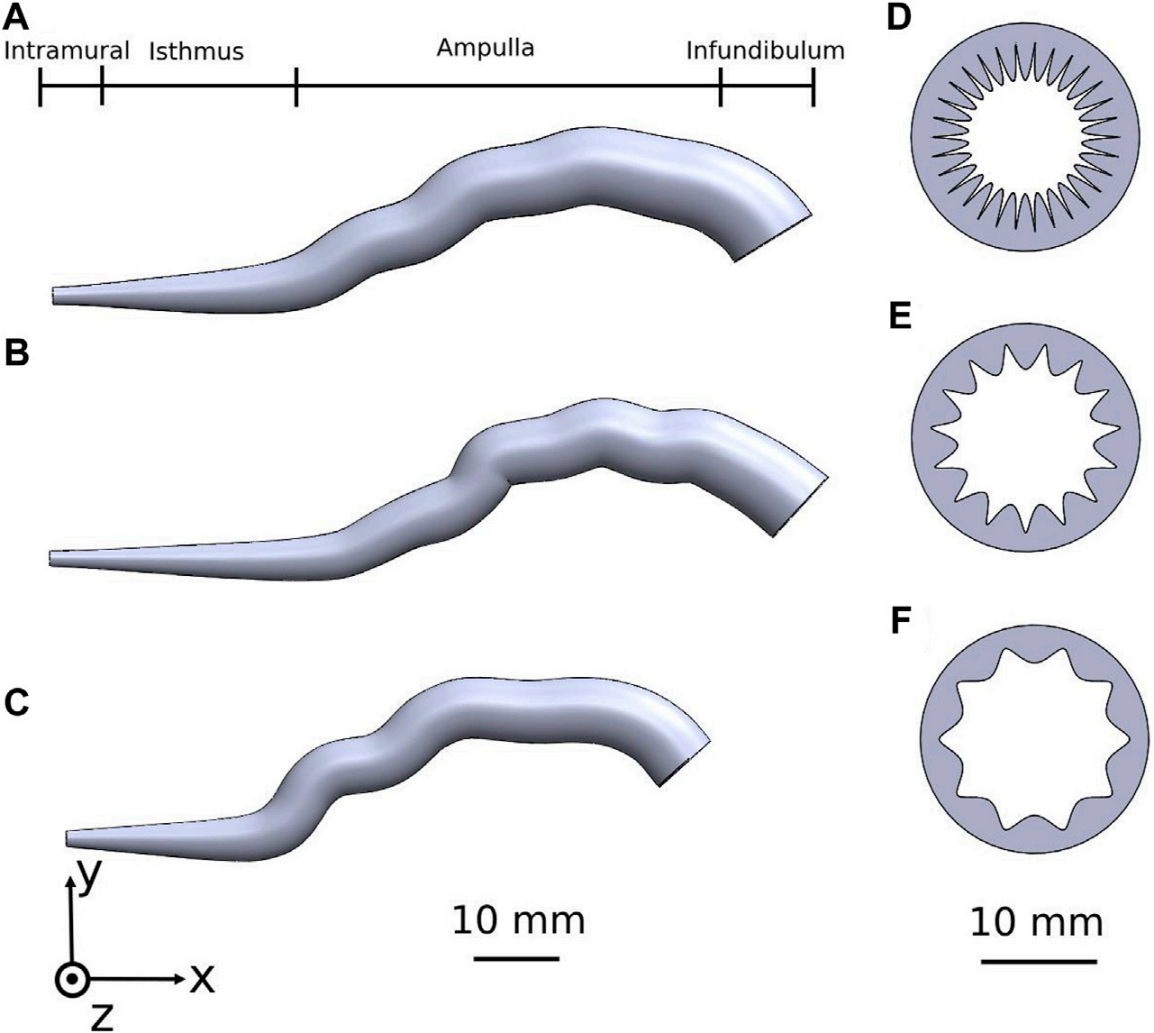
Human fallopian tube geometrical 3D models of women at their: **(A)** 20s, **(B)** 30s, and **(C)** 40s. The coinciding fallopian tube cross-sections illustrating the internal longitudinal folds of the mucosal layer are shown in **(D–F)**.

We designed and constructed three detailed 3D human fallopian-tube models: one for women ages in the middle 20s, one for women ages in the middle 30s, and one for women ages in the middle 40s (Figure 1). We built the different geometrical fallopian tubes based on Magnetic Resonance Imaging (MRI) results of the human fallopian tubes (Rimbach et al., 1998; De Geyter et al., 2007; La Parra Casado et al., 2013; Revzin et al., 2020; Luo et al., 2023) and based on studies about the morphological changes in the human uterine tube according to aging (Lisa et al., 1954; Kim-Bjorklund et al., 1991; Hwang and Song, 2004; Wolman, 2014; Talukdar and Sahu, 2016; Devi et al., 2017; Veerraju and Sesi, 2019). Each 3D geometrical model is considered a finite three-dimensional elongated trumpet-shaped structure crowded with ciliated cells. The fallopian tube model is approximately a narrow tube, 88–105 mm in length and 5–12 mm in diameter with radial tapering in the intramural portion.

**FIGURE 2:**
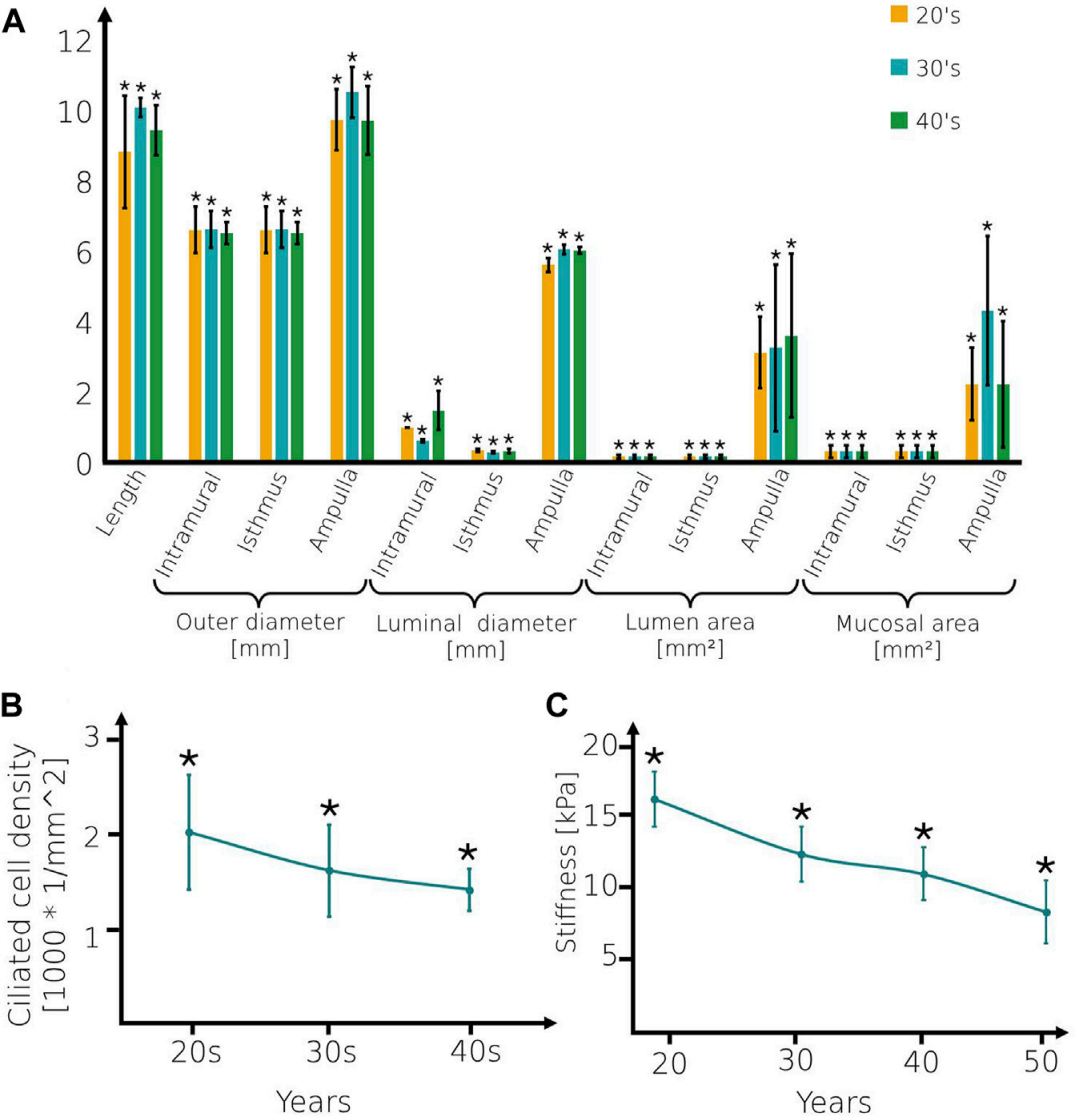
Geometrical and mechanical values of the fallopian tube models. **(A)** The geometrical parameters, **(B)** Ciliated cell density of fallopian tube models, and **(C)** Stiffness of the mucosal layer for women at their 20s, 30s, and 40s. Bars are mean ± SEM (standard error of the mean). (*) Significantly different from the normal control group (*p* < 0.05).

The 3D models are composed of four regions: intramural, isthmus, ampulla, and infundibulum (Figure 1). The isthmus and intramural are the straight part of the fallopian tube and approximately 40 mm in length and 5–7 mm in diameter. The ampulla, a highly ciliated central portion, is a convoluted site approximately 50 mm long and 8–11 mm in diameter. The inner surface of the fallopian tube model is protruded by internal longitudinal folds with ciliated cells, presenting the mucosal layer. The folds of the mucosal layer change from columnar to cuboidal shape according to the different age groups (see Figures 1D–F). The tubal fluid flows inside the fallopian tube model in the lumen area and helps the sperm cells reach the ampulla where fertilization occurs. The geometrical parameters of each part of the fallopian tubes are varied according to the women’s ages (Figure 2A) (Lisa et al., 1954; Kim-Bjorklund et al., 1991; Hwang and Song, 2004; Talukdar and Sahu, 2016; Veerraju and Sesi, 2019). The models were built in SolidWorks (Premium 2022 x64 Edition, Waltham, MA, USA).

### 2.2 Peristaltic-ciliary

Ciliated cells are dispersed on the longitudinal folds of the mucosal layer. Here, we built 3D flexible cylinders connected with the mucosal layer, presenting the axonemal structure of cilia. The ciliated cell concentration decreases as the woman ages (Li et al., 2013; Tao et al., 2020) (see Figure 2B). Here, we applied uniformly centripetal force on each 3D cilia model as a function of time. The direction of the force is orthogonal to the cilia and toward the center of the curvature. We applied a force magnitude of $0.42*10^{-17}\left[µN\right]$ to achieve the ciliary beat frequency reported in previous studies, which is 5.4 Hz (Lyons et al., 2002; Papathanasiou et al., 2008). Supplementary Video S1 shows a single 3D cilium beating and indicates the magnitude and directions of the applied forces. We assumed that the mucosal layer is made of isotropic compressible materials and used a Neo-Hookean constitutive model with strain energy function provided in Equations (1), (2), and (3):

$$W=\frac{\mu }{2}\left(I_{1}-3\right)+\mu \ln J+\frac{\lambda }{2}{\left(\ln J\right)}^{2},$$ (1)

$$\lambda =\frac{\nu L*\kappa }{\left(1+\nu \right)\left(1-2\nu \right)*A},$$ (2)

$$\mu =\frac{L*\kappa }{2A\left(1+\nu \right)},$$ (3)

where $I_{1}$ is the first invariant of the right Cauchy-Green deformation tensor, J is the determinant of the deformation gradient tensor. The parameters μ and λ are the Lamé parameters that relate to stiffness of the mucosal layer, L is the length, Ais the luminal area, and ν=0.49 is the Poisson’s ratio of the muscle layer (Baah-Dwomoh et al., 2016; Singh and Chanda, 2021) (Figures 2A–C). Supplementary Video S2 shows the cilia beat in the mucosal layer of the 3D fallopian tube model for women at their 20s.

### 2.3 3D human sperm models

**FIGURE 3:**
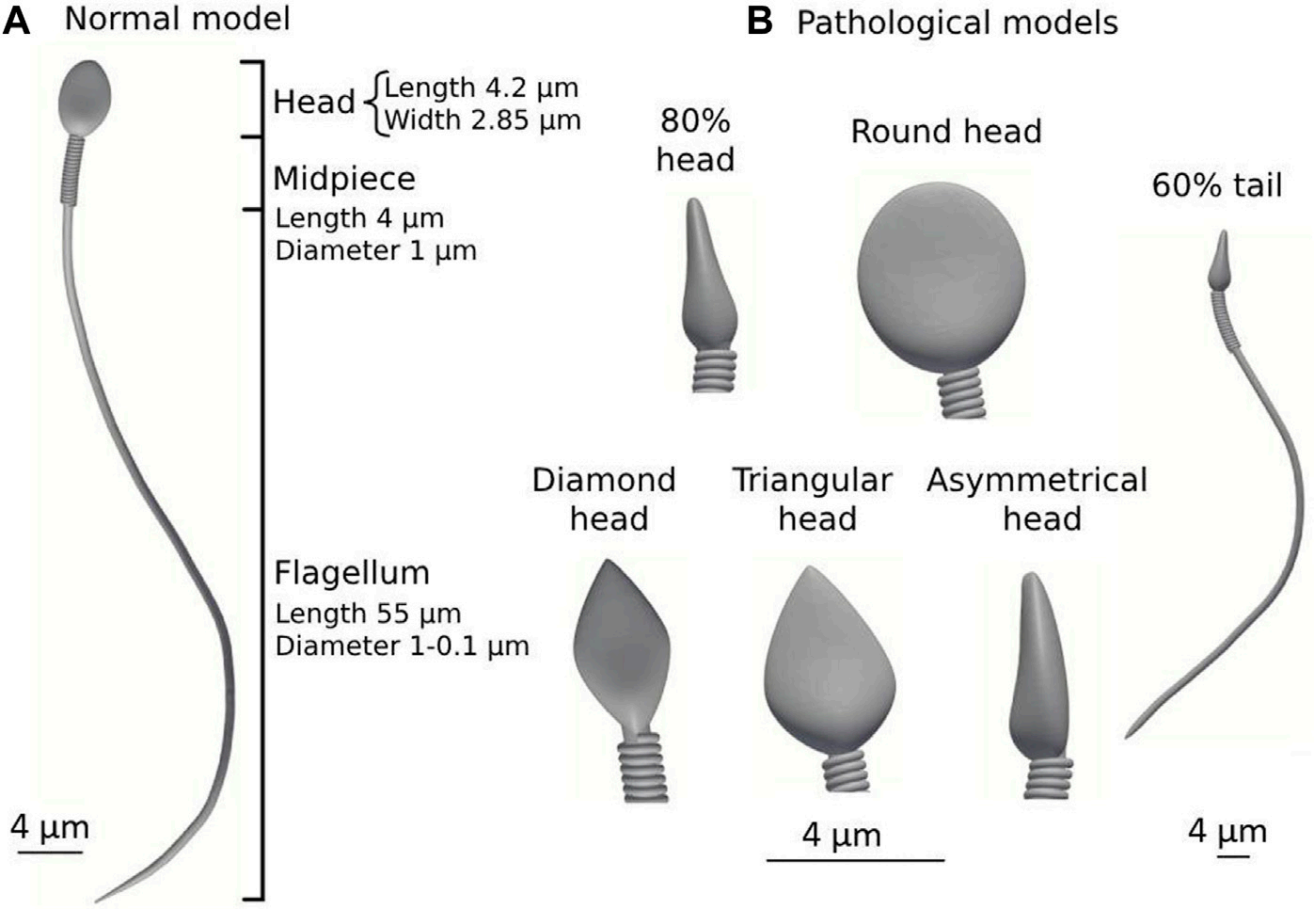
Normal and pathological sperm cell models (according to the WHO classification (World Health Organization, 2021)). **(A)** Normal sperm cell model. **(B)** Different pathological sperm cell models.

We used the 3D geometrical models of the normal and abnormal sperm cells developed in our previous study (Nassir et al., 2022; 2023). We built a numerical mechanical sperm model based on previous dynamic 3D optical imaging experimental results of sperm cells done by our group (Dardikman-Yoffe et al., 2020). Shortly, a normal-morphology sperm cell swimming in the fluid was acquired with our dynamic interferometric optical computer tomography method at a spatial resolution of 0.5 microns, achieving both retrievals of the 3D refractive index profile of the sperm head and the detailed four-dimensional localization of the thin, highly dynamic sperm flagellum. To allow processing many of cell’s swimming together, we simplified the 3D geometry of the sperm models developed in our previous study so that the models possess full sperm geometry with only the external sperm cell real organelles (head, flagellum, and midpiece). The head of the model is approximated by an elliptical shape, 4.2 µm long and 2.85 µm wide with radial symmetrical tapering reaching the tip of the head, approximated as 0.46 µm. The midpiece contains a filament structure surrounded by spiral arrays of mitochondria, 4 µm in length and 1 µm in diameter. The flagellum is comprised of a 3D flexible filament with a length of 55 µm and its diameter decreases from 1 μm to 0.1 μm at the distal end. Dynein motors proteins are located along the tail filament (Lindemann and Lesich, 2016). Here, we tested 7 detailed dynamic 3D sperm cell models: one for a normal sperm cell and 6 for abnormal morphology sperm cells with different types of abnormalities. These include defects in morphology of the tail (short tail), head size (small head), and head shape (triangular, round, asymmetrical, and diamond heads) (Nassir et al., 2023) (Figure 3).

### 2.4 Beat pattern

The developed model is a mechanical, mathematical model, based on kinetic laws and finite elements’ motion in 3D. Each element of the model is described as a node in a mesh and treated as an independent body, based on the finite-elements theory. Forces are applied to the elements, each of which moved in the 3D space creating its 3D path. The composition of all elements describes the path of the cell. Each dynein component is designated to be an element, sliding based on its curvature and position change. The axonemal beating is created by activating the dynein motors located on the single filament of the overall flagellum. Using Frenet equations, we converted the applied forces to position and rotation changes for each element. Finally, we used the location and rotation changes to create a simulation of the beating pattern of the sperm cell. The number of active dynein motors along the filament is determined to be *N* = 100 nodes. The beating patterns of the different models are unaffected by larger numbers of nodes but influence the simulation runtime and memory. Low simulation resolutions are obtained with fewer than 100 nodes of dynein motors. The normal and abnormal sperm models were considered to be hyperactivated sperm models. The 3D hyperactivated beating patterns of the different models were created by increasing the dynein sliding forces applied to the sperm models from the previous study (Nassir et al., 2023).

The 3D geometry of the normal and pathological sperm cell models was simplified to optimize the complex dynamic simulations. The optimized simulation includes a large number of swimming sperm cell models, short runtimes, saving memory, and high resolution. For verification, we performed a flagellum beating comparison between the full model and the simplified model by presenting the movement characteristics of the sperm models such as the flagellar amplitude, flagellar beat frequency, and straight-line velocity (see Supplementary Video S3).

### 2.5 Computerized fluid-dynamic simulation

We studied sperm swimming inside a viscous medium in the different 3D fallopian tube models. This viscous medium is considered to be a fluid that has the same thermo-physical properties as the cervical mucus to imitate the biophysical environment mimic system of the human fallopian tubes such as density $1007\;\left[\frac{\text{kg}}{{\text{mm}}^{2}}\right]$ , specific heat $4140\;\left[\frac{J}{\text{kJ}*K}\right]$ , thermal conductivity $0.627\;\left[\frac{W}{\text{mK}}\right]$ , and dynamic viscosity $\left(0.02\gamma +0.98\right)\left[\text{Pa}*\sec\right]$ (Lai et al., 2007; Lafforgue et al., 2017; Nassir et al., 2023). We solved the Navier-Stokes equations to describe the flow of the cervical mucus in the 3D fallopian tube model. To represent the Navier-Stokes equations, we used Computerized Fluid Dynamic (CFD) analysis software by SolidWorks flow simulation. These equations are described by formulations of mass, momentum, and energy conservation laws [see Equations (4), (5), (6), and (7)] (Sobachkin and Dumnov, 2013; Jonuskaite, 2017):

$$\frac{\partial \rho }{\partial t}+\frac{\partial \left(\rho u_{i}\right)}{\partial x_{i}}=0,$$ (4)

$$\frac{\partial \left(\rho u_{i}\right)}{\partial t}+\frac{\partial }{\partial x_{j}}\left(\rho u_{i}u_{j}\right)+\frac{\partial P}{\partial x_{i}}=\frac{\partial }{\partial x_{j}}\left(\tau_{ij}+\tau_{ij}^{R}\right)+S_{i},$$ (5)

$$\frac{\partial \rho E}{\partial t}+\frac{\partial \rho u_{i}\left(E+\frac{P}{\rho }\right)}{\partial x_{i}}=\frac{\partial }{\partial x_{i}}\left(u_{j}\left(\tau_{ij}+\tau_{ij}^{R}\right)+q_{i}\right)-\tau_{ij}^{R}\frac{\partial u_{i}}{\partial x_{j}}+\rho \varepsilon +S_{i}u_{i},$$ (6)

$$E=e+\frac{u^{2}}{2},\tau_{ij}=µS_{ij},\tau_{ij}^{R}={µ}_{t}S_{ij}-\frac{2}{3}\rho \kappa \delta_{ij},S_{ij}=\frac{\partial u_{i}}{\partial x_{j}}+\frac{\partial u_{j}}{\partial x_{i}}-\frac{2}{3}\delta_{ij}\frac{\partial u_{k}}{\partial x_{k}}$$ (7)

where ρ is the fluid density, t is the time, u is the velocity, P is the pressure, τ is the stress tensor, q is the heat flux, ε−κ is the turbulent model and µ is the fluid viscosity.

To perform dynamic simulations, we meshed the 3D geometrical sperm models. We used the generation and processing toolbox by SolidWorks to create a 3D tetrahedral element mesh for this purpose. The mesh densities of the different models vary between the cells. This variability can affect the simulation runtime but negligibly changes the outcome measures (less than 3%). The models were assumed to be hyper-elastic uncompressible materials with the Neo-Hookean constitutive model. The models were assumed to be uncompressible materials with uniform mechanical proprieties for all components, such as the density. Since the cell head is the major component by weight, changing the density of the components is not expected to significantly affect the swimming behavior (Nassir et al., 2023).

We applied a dynamic simulation for each fallopian tube model. We analyzed the beating patterns of the normal and pathological sperm cell models swimming inside a viscous fluid in each 3D fallopian tube model. For each fallopian tube model, we simulated 10,000 sperm models with the same morphology, in total 70,000 sperm models for each simulation. The sperm models were assigned a random initial position within the intramural portion and were free to swim through the 3D fallopian tube model. The dynamic simulations were performed using SolidWorks 2022, Blender 2.91, and Python 3.3 SW (3D modelling and rendering package).

### 2.6 Outcome measurements

The 3D swimming trajectories of the different models were analyzed for position maps. The localized displacements that developed in the sperm head centroid of the normal and pathological models were determined as a function of time. The results were calculated by SolidWorks 2022, Blender 2.91 Python 3.3 SW (3D modelling and rendering package). Outcome measures included:

1. The 3D swimming trajectories of the sperm head centroid of the normal and pathological models, across the different 3D human female fallopian tube models over 5 h.
2. The quantity of the normal and pathological sperm models reaching the ampulla portion where the fertilization occurs.
3. The velocity of the normal and pathological sperm models inside the different 3D human female fallopian tube models.
4. Evaluation of the selection ability of the 3D fallopian tube model with age by comparison between the percentages of the normal and pathological sperm cells inside the ampulla portion.

## 3 Results

**FIGURE 4:**
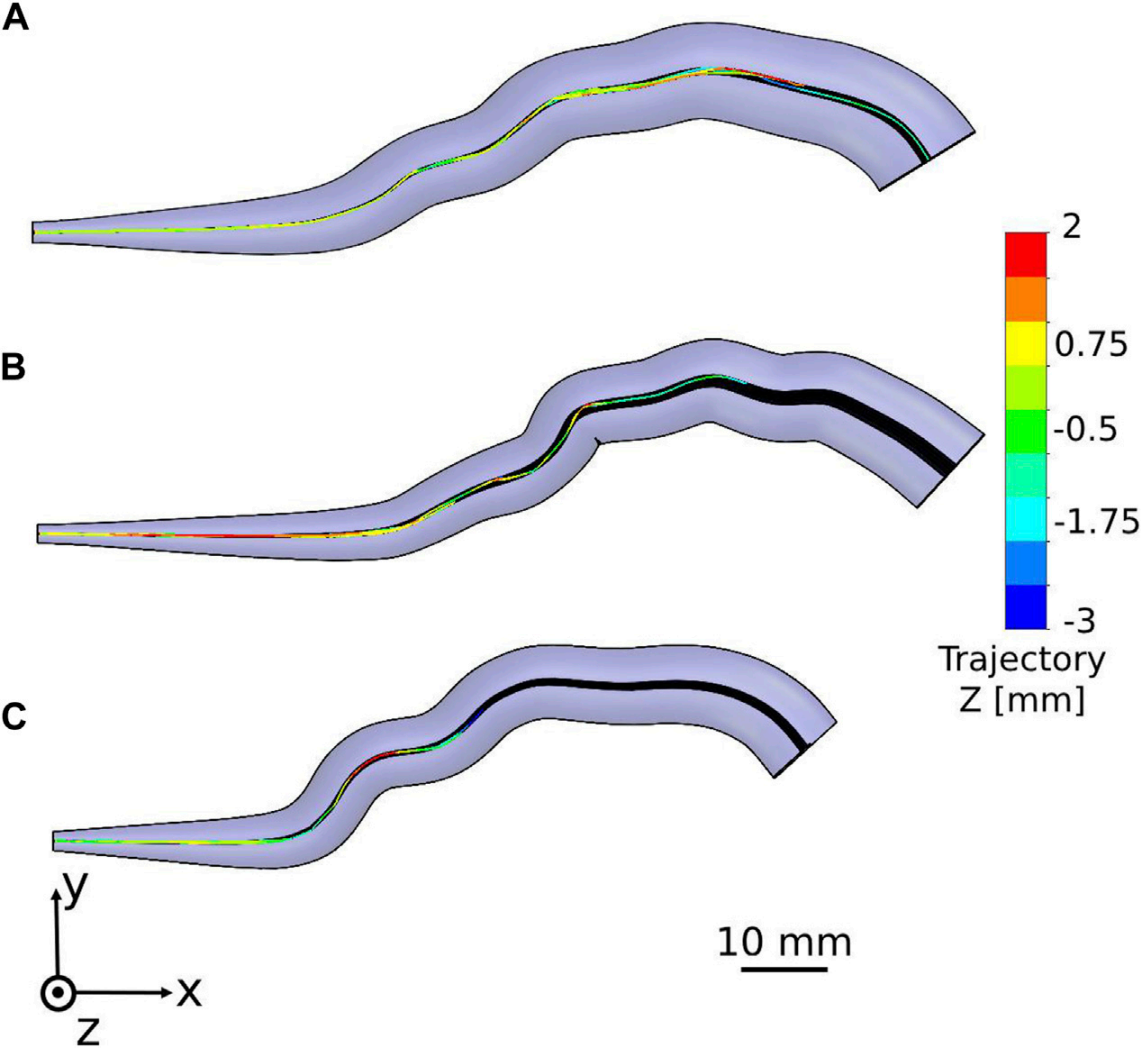
Swimming patterns of the normal sperm models in the different fallopian tube models. Two-dimensional trajectories in the anterior view of the fallopian tube model for women at their **(A)** 20s, **(B)** 30s, and **(C)** 40s.

We tracked the sperm head centroid of each model during swimming in the different fallopian tube models. The 2D trajectories of the normal sperm models through each fallopian tube model are shown in Figure 4. For all ages, we observed that the normal sperm models moved forward inside the fallopian tube with a small lateral displacement. This swimming behavior is equivalent to the swimming pattern of the normal sperm cells reported in previous bio-mechanical, microfluidic, and experimental imaging studies of human sperm cell motility (Chenoweth, 2005; Su et al., 2012; Sartori et al., 2016; Rode et al., 2019; Dardikman-Yoffe et al., 2020; World Health Organization, 2021; Nassir et al., 2022, 2023). Figure 4 shows that most of the normal sperm models accumulated in the intramural and isthmus regions due to the small luminal area. Some of the normal sperm models kept swimming near the fallopian tube walls and toward the fertilization site, the ampulla. The number of the normal sperm models that succeeded to pass to the ampulla is the largest in the fallopian tube of women at their 20s. In addition, at the 20s, the normal sperm models swam to greater distances inside the ampulla compared to older ages. Hence, as a woman’s age increases fewer normal cells succeed in reaching the ampulla region; moreover, they swim for shorter distances toward the ovary (Figure 4).

**FIGURE 5:**
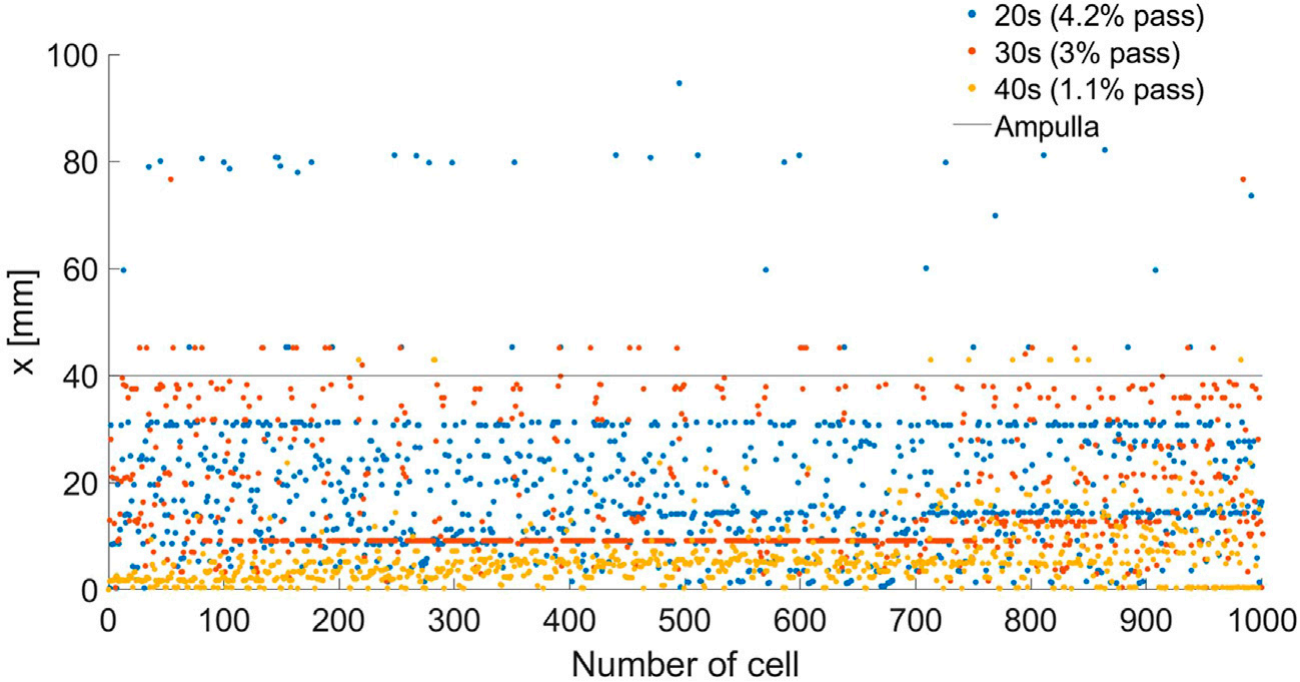
Final depths (*x*) inside the woman’s body of 1000 normal sperm models in the forward direction inside the different fallopian tube models. The percentages of normal sperm models that reach the ampulla region are 4.2%, 3%, and 1.1% for women at their 20s (blue dots), 30s (red dots), and 40s (yellow dots), respectively.

We described the positions of the normal sperm models inside the different 3D fallopian tube models after 5 h. We evaluated the fallopian tubes based on the numbers and the positions of the normal sperm models accumulated in the isthmus or reached the fertilization site, the ampulla. After 5 h, all the sperm models remained in their local location inside each fallopian tube model. Figure 5 describes the final positions of 1000 normal sperm models in the forward direction inside each fallopian tube. For all ages, most normal sperm models accumulated in the isthmus region and were unable to continue swimming into the ampulla. For women at their 20s, 4.2% of the normal sperm models succeeded in passing the isthmus region and continued to swim inside the ampulla. The percentages of normal sperm models that reached the ampulla decreased with age, 3% for women at their 30s and 1.1% for women at their 40s. With age, the normal sperm models did not get as far inside the ampulla, which can affect their chances to reach the egg. For women at their 20s, the normal sperm models swam approximately 40 mm into the ampulla, whereas they swam about 30 mm for women at their 30s and 10 mm for women at their 40s.

**FIGURE 6:**
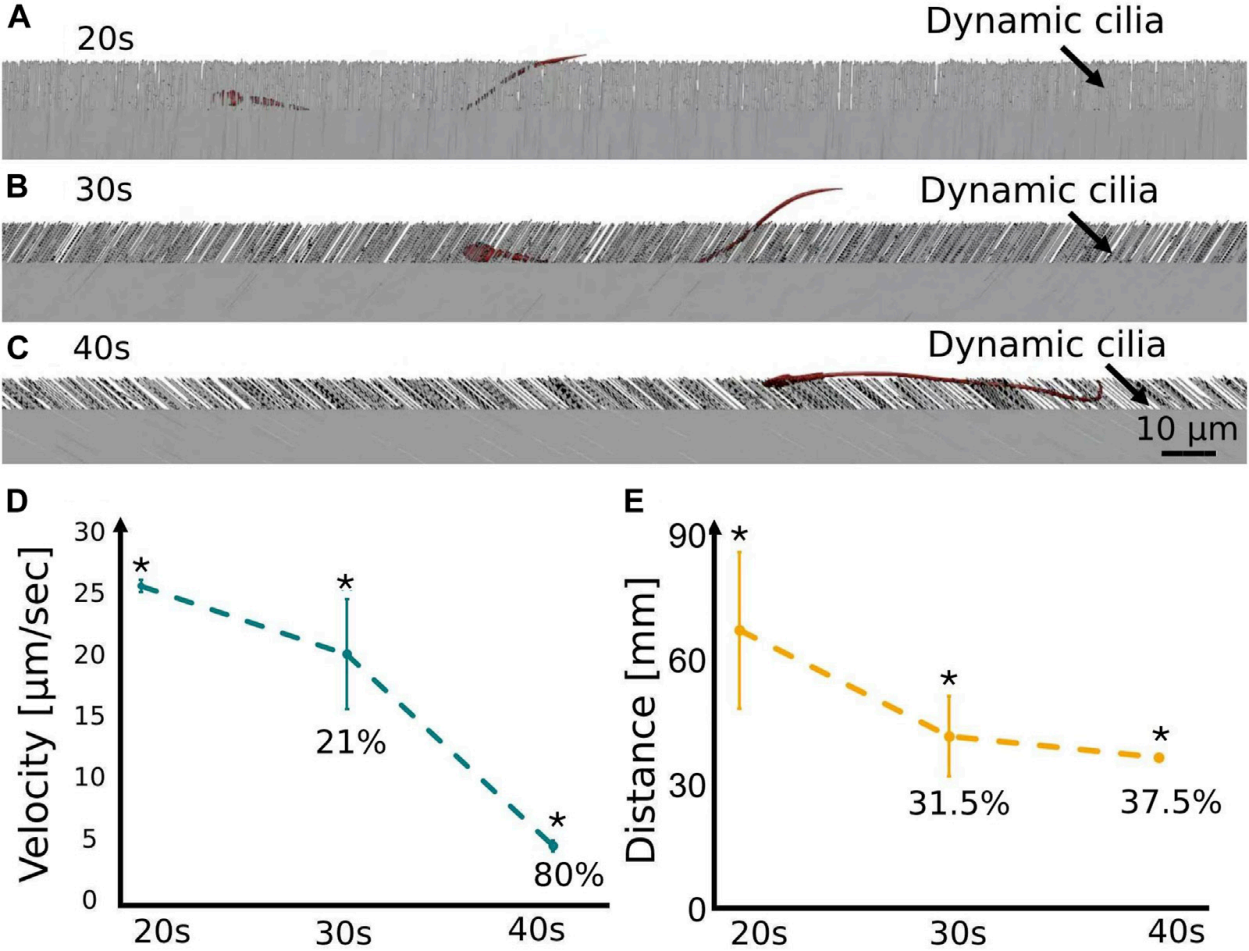
Swimming behavior of the normal sperm models in the different fallopian tube models. A single normal sperm model swims in the 3D fallopian tube model for women at their **(A)** 20s, **(B)** 30s, and **(C)** 40s. The average velocity **(D)** and the average distance **(E)** of the normal sperm models through the 3D fallopian tube models, at women at their 20s, 30s and 40s (see Supplementary Video S4). Bars are mean ± SEM (standard error of the mean). (*) Significantly different from the normal control group (*p* < 0.05).

Sperm cell motility is a significant parameter for successful fertilization. The sperm cells’ velocity and swimming distance inside the fallopian tube are affected by the morphological and mechanical changes in the fallopian tube due to women aging. To evaluate the woman’s age influence on sperm cell motility, we calculated the average velocities and swimming distances of the normal sperm models through the 3D fallopian tube models for women at their 20s, 30s and 40s. Figures 6A–C show the positions of a single normal sperm model inside the different fallopian tube models. We calculated the average velocities and distances for all the sperm cells inside the fallopian tube models and calculated the standard error of the mean (SEM) for each women’s age group, each group contains 100 experiments. We observed that for women at ages 20s the normal sperm models had the highest average velocity ( $24.43\;\pm 0.46\;\frac{µm}{\sec}$ ) and the highest average swimming distance inside the fallopian tube $\left(68.8\;\pm 15.9\text{ mm}\right)$ , compared with the other age groups. The average velocity of the normal sperm models swimming in the fallopian tube is $19.29\;\pm 4.12\;\frac{µm}{\sec}$ for women at their 30s, and $\left(4.88\;\pm 0.42\;\frac{µm}{\sec}\right)$ for women at their 40s. The average swimming distance of the normal sperm models in the fallopian tube is $\left(47.1\;\pm 8.18\text{ mm}\right)$ for women at their 30s, and $\left(43\;\pm 0.1\text{ mm}\right)$ for women at their 40s. Normal sperm models velocity inside the fallopian tube decreased by 21% and 80%, at the 30s and 40s, respectively, compared to the velocity for women at their 20s (see Figure 6D). Normal sperm models swimming distance inside the fallopian tube decreased by 31.5% and 37.5%, at the 30s and 40s, respectively, compared to the velocity for women at their 20s (see Figure 6E). Hence, woman aging has a significant effect on sperm cell velocity and swimming distance, which can influence their journey to meet the egg in the ampulla region (see Supplementary Video S4).

**FIGURE 7:**
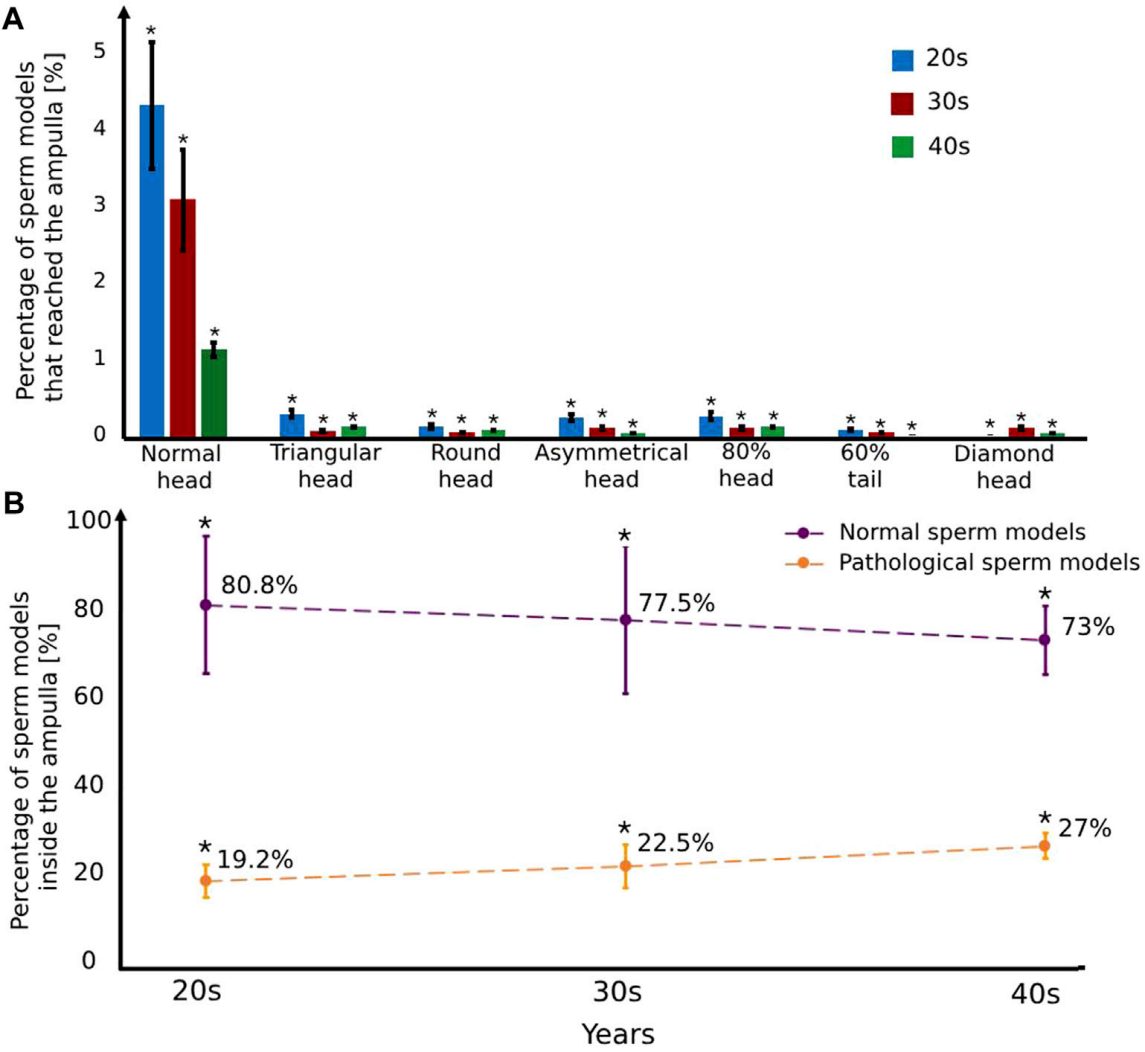
The percentage of the normal and pathological sperm models that reached the ampulla versus various typical sperm morphologies **(A)** and women’s age groups **(B)**. Bars are mean ± SEM (standard error of the mean). (*) Significantly different from the normal control group (*p* < 0.05).

We calculated the percentages of the normal and pathological sperm models that succeeded in reaching the ampulla of each age group fallopian tube model (Figure 7). Most of the sperm cell models accumulated in the isthmus region due to a smaller luminal area. For all ages, the percentages of normal sperm models that enter the ampulla region are the highest (1.1%–4.2%), compared to the pathological sperm models (0%–0.3%) (Figure 7A). The pathological sperm models swam inside the small luminal area in 3D lateral displacement and twisted trajectories; therefore, few pathological sperm models cross the isthmus region and reach the fertilization site, the ampulla (see Figure 7A). We evaluated the selection ability of the different fallopian tubes by comparison between the percentages of the normal and the pathological sperm models inside the ampulla area (see Figure 7B). For women at their 20s, 80.8% ± 15.4% (*n* = 10,000 sample tested) of the total sperm models that reached the ampulla are normal sperm models and 19.2% ± 3.6% (*n* = 10,000 sample tested) of them are abnormal sperm models. With age, the percentage of the normal sperm models inside the ampulla decreases, 77.5% ± 16.5% (*n* = 10,000 sample tested) for women at their 30s and 73% ± 7.7% (*n* = 10,000 sample tested) for women at their 40s. On the other hand, with age, the percentage of the pathological sperm models inside the ampulla increases, 22.5% ± 4.8% (*n* = 10,000 sample tested) for women at their 30s and 27% ± 2.8% (*n* = 10,000 sample tested) for women at their 40s (see Figure 7B). The decrease in the percentage of normal sperm cells and the increase in the percentage of pathological ones inside the ampulla due to aging can decrease the chances of normal sperm cells succeeding in reaching the egg and fertilizing it.

**FIGURE 8:**
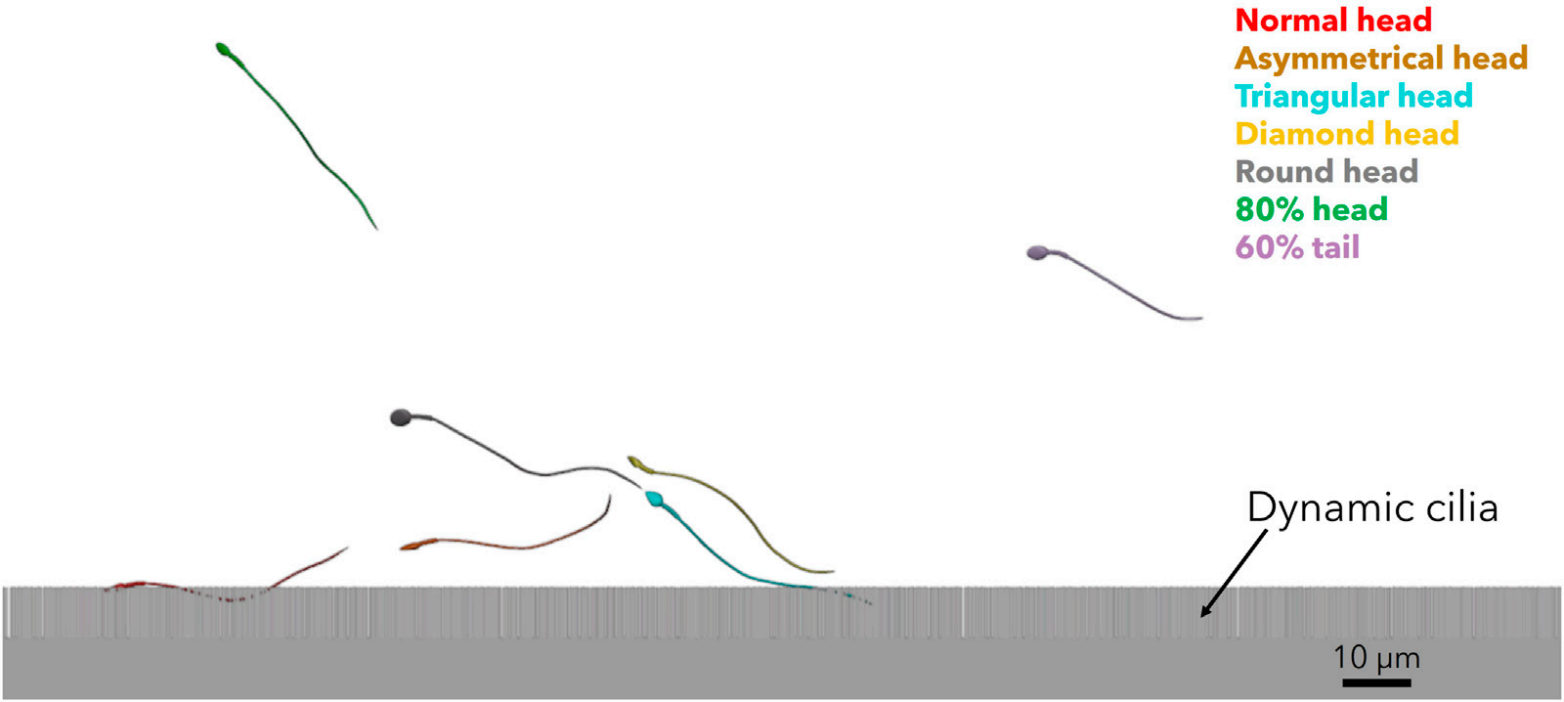
3D sperm cells swimming near the mucosal layer of the fallopian tube model for women at their 20s (see Supplementary Video S5).

We tracked the sperm models during swimming near the mucosal layer of the different fallopian tube models. For all ages, we observed that most normal sperm models prefer to swim beside the fallopian tube walls, in contrast to the pathological sperm models that swim inside the luminal area with 3D lateral displacements. Figure 8 and Supplementary Video S5 show the position of a single sperm model of each morphology in the fallopian tube model for women at their 20s. The swimming section shown in Figure 8 and Supplementary Video S5 only describes a local path of the overall swimming behaviour for each sperm model. The 3D swimming patterns were affected by the collision angle with the surface, collision velocity, cell-to-cell interactions, and the local geometry of the collision site. The swimming behavior of the sperm cells near the surface is equivalent to previous studies that tracked the healthy sperm cells swimming close to walls (Chenoweth, 2005; Su et al., 2012; Dardikman-Yoffe et al., 2020; World Health Organization, 2021; Nassir et al., 2022; Nassir et al., 2023).

## 4 Discussion

In this study, we evaluated the women’s aging effect on the chances of sperm cells to reach the fertilization site by describing their swimming behavior. This was done for three typical morphologies describing the human fallopian tube over age. Using a unique combination of simulation tools, we implemented 3D detailed geometrical models of normal and pathological sperm cells swimming together in 3D models of three human fallopian tubes based on human uterus. We also applied peristalsis-ciliary activity along the inner walls of the different fallopian tube models. Dynamic simulations were performed for the different human sperm models swimming inside the fallopian tube models to evaluate the women’s aging influence on the changes of sperm cells of reaching the egg. The fallopian tubes provide a proper environment for sperm cells and oocyte transportation and have a significant influence on fertility. The fallopian tube undergoes morphological and mechanical changes due to women’s aging, which may impair fertility. Our study shows that this also happens due to impairing the ability of sperm cells to reach the fertilization site of the aging woman. Thus, this advanced mechanical model of the swimming behavior of the sperm cells inside the fallopian tube of different women’s age groups can play a role key in understanding the women’s aging influence on fertility.

For all age groups, we observed that the normal sperm models moved forward inside the fallopian tube models with a small lateral displacement. Most of the 3D trajectories of the normal sperm models were close to the fallopian tube walls near the internal longitudinal folds of the mucosal layer. Our findings match the results of previous studies about tracking healthy human sperm cells (Chenoweth, 2005; Su et al., 2012; Dardikman-Yoffe et al., 2020; World Health Organization, 2021; Nassir et al., 2022; Nassir et al., 2023) and sperm cell-to-surface interactions (Guidobaldi et al., 2015; Rode et al., 2019; Zaferani et al., 2021). For all age groups, most of the normal sperm models accumulated in the intramural and isthmus regions due to the small luminal area. Swimming behavior characteristics through a narrow path such as linear and angular velocity, swimming directions, and distances can be affected due to the increase in the cell-to-cell and cell-to-surface interactions. Therefore, few normal sperm models succeeded in passing the narrow isthmus region and continued swimming into the ampulla. These results are equivalent to those of previous studies that considered the narrow paths as a significant barrier and selector against sperm cells (Bartoov et al., 1982; Denissenko et al., 2012; Heydari et al., 2023).

The number of the normal sperm models in the fertilization site, the ampulla, decreases with the women’s aging. Furthermore, with women’s aging, the normal sperm cells swim for shorter distances inside the ampulla toward the ovary, and the empty area from normal sperm cells inside the ampulla increases, which lowers the success chances of the sperm cell reaching the oocyte. The aging of the human fallopian tube also affects the average velocity of normal sperm cells. Low sperm cell motility, especially inside the fertilization site of the fallopian tube has a significant impact on fertility impairment (Hunter, 1998; Ahlgren, 2010; Ambildhuke et al., 2022). Thus, women’s aging is associated with a decrease in the quantity, swimming distance, and velocity of the normal sperm cells inside the fertilization site due to the morphological and mechanical changes in the fallopian tube over aging. The changes that the fallopian tube undergoes because of women’s aging, such as the small luminal area, low internal longitudinal folds, and low wall stiffness, prevent most of the normal sperm cells from moving inside the fallopian tube toward the fertilization site. The cell-to-cell and cell-to-surface collisions increase in smaller swimming luminal areas; therefore, fewer normal sperm cells can cross the isthmus region and reach the ampulla. The normal sperm models swam close to the internal longitudinal folds inside the different fallopian tube models. With age, the folds surface decreases due to the low quantity of internal longitudinal folds. Hence, the normal sperm models swim in smaller swimming areas of the fold surfaces as the age increases. This small swimming area also causes more collisions and interruptions against the sperm cells’ progress forward. Furthermore, the stiffness of the internal wall of the fallopian tube decreases with women’s aging. Therefore, the friction between the normal sperm models and the internal wall increases and decelerates the velocity of the normal sperm models toward the ovary. Our study thus confirms the results of previous studies in this field about the defects in function and selection ability of the human fallopian tube due to women’s aging (Pinero and Foraker, 1963; Bouyer et al., 2003; Maheshwari et al., 2008; Briceag et al., 2015; Szmelskyj et al., 2015).

Lastly, we observed that for all age groups the number of normal sperm models that succeeded in reaching the ampulla is larger compared with the pathological sperm models. On the other hand, the percentage of normal sperm models inside the ampulla decreases with aging while the percentage of the pathological sperm models increases. Most normal sperm models prefer to swim beside the fallopian tube walls, in contrast to the pathological sperm models that swim inside the luminal area with 3D lateral displacements. The main morphological and mechanical changes in the fallopian tube due to aging occur in the internal mucosal walls; therefore, the swimming behavior of the normal sperm models is more affected than the swimming behavior of the pathological sperm models. As the percentage of pathological sperm cells increases in the ampulla, the success chances of a normal sperm cell reaching the egg and fertilizing it are statistically lower with age. With age, the normal sperm cells encounter more difficult challenges in their journey inside the fallopian tube and their success chances of reaching the fertilization area and the egg diminishes.

Note that this study focuses on hyperactivated sperm within the fallopian tube due to its crucial role in the fertilization process and its potential implications for reproductive success, especially concerning women’s aging. Hyperactivated sperm display heightened motility patterns linked to successful fertilization, facilitating better penetration of the oocyte’s protective barriers. By centering on hyperactivated sperm, the study focuses on how age-related alterations in the fallopian tube environment can affect sperm behavior and fertility. The emphasis on hyperactivated sperm in this study does not diminish the importance of examining other motility patterns. The study is the initial step for future research of analyzing the entire range of sperm behavior within the fallopian tube.

Our study builds upon empirical data from prior research on gamete behavior within the female reproductive system. It validates different stages of our simulations and elucidates how such clinical outcomes can be achieved through a detailed mechanical and computer model, which is impractical to perform in an actual clinical trial on humans. We address this gap and offer overall simulative tools for impactful conclusions that can enhance our understanding of sperm cell behavior within the female reproductive system and its implications for fertilization chances. To conclude, we have presented an advanced dynamic mechanical model for evaluating the women’s aging influence on the motility of healthy and pathological human sperm cells inside the female fallopian tubes. Our model describes the impact of the morphological and mechanical changes in the fallopian tube on fertilization potential due to women’s aging. Our method combines full dynamic 3D modeling of different human sperm cells, free swimming inside different 3D fallopian tube tracts, and interactions with real internal environment parameters. This method may lead to additional studies in evaluating the age influence on the selection ability of the fallopian tubes and provide missing links in previous studies. Especially, our study on sperm cell motility through the fallopian tube in relation to different ages provides a new scope for investigation and treatment of diseases and infertility cases associated with aging. Moreover, this new approach can be the basis for personalize medicine in fertility treatments, as it can simulate the chances of a certain woman to conceive from a certain sperm sample; thus being able to adapt and select certain sperm features to certain woman reproductive organs and age and use them for intrauterine insemination (IUI).

## Data Availability

The original contributions presented in the study are included in the article/Supplementary Material, further inquiries can be directed to the corresponding author.

## Supplementary material

SUPPLEMENTARY VIDEO S1 3D Beating of a Single Cilium, Indicating the Associated Forces.

SUPPLEMENTARY VIDEO S2 The Cilia Beat in the Mucosal Layer of the 3D Fallopian Tube Model for Women at their 20s.

SUPPLEMENTARY VIDEO S3 Demonstration of Free Swimming of the Full and Simplified Sperm Models.

SUPPLEMENTARY VIDEO S4 Normal Sperm Models Swim in the Different 3D Fallopian Tube Models.

SUPPLEMENTARY VIDEO S5 3D Sperm Cells Swimming Near the Mucosal Layer of the Fallopian Tube Model for Women at their 20s.
